# Nutraceutical Effect of Resveratrol on the Mammary Gland: Focusing on the NF-κb /Nrf2 Signaling Pathways

**DOI:** 10.3390/ani13071266

**Published:** 2023-04-06

**Authors:** Muhammad Umair Ul Hassan Malik, Nighat Hashmi, Marium Khan, Zain ul Aabdin, Rokayya Sami, Amani H. Aljahani, Rasha A. Al-Eisa, Mamdoh S. Moawadh, Naseh A. Algehainy

**Affiliations:** 1 Punjab Medical College, Faisalabad 38000, Pakistan; 2 Rural Health Center Garh Maharaja, Tehsil Ahmed PurSial, Jhang 35080, Pakistan; 3 Quaid-e Azam Medical College, Bahawalpur, Affliated University of Health Sciences Lahore Punjab, Lahore 54600, Pakistan; 4 Department of Preventive Veterinary Medicine and Public Health, Faculty of Veterinary and Animal Sciences, Ziauddin University, Link Road Campus Education City Kathore, Karachi Sindh 75000, Pakistan; 5 Department of Food Science and Nutrition, College of Sciences, Taif University, P.O. Box 11099, Taif 21944, Saudi Arabia; 6 Department of Physical Sport Science, College of Education, Princess Nourah bint Abdulrahman University, P.O. Box 84428, Riyadh 11671, Saudi Arabia; 7 Department of Biology, College of Sciences, Taif University, P.O. Box 11099, Taif 21944, Saudi Arabia; 8 Department of Medical Laboratory Technology, Faculty of Applied Medical Sciences, University of Tabuk, Tabuk 71491, Saudi Arabia

**Keywords:** lipopolysaccharides, nutraceutical, inflammation, resveratrol, mammary gland

## Abstract

**Simple Summary:**

It is essential to study animal models of human disease to understand the progression of disease, the mechanisms involved and their interventional testing on a pre-clinical basis. We proposed to observe a lipopolysaccharide (LPS)-induced effective model of mammary gland inflammation. Additionally, the nutraceutical role of resveratrol, a phenolic compound, against the negative effects of lipopolysaccharides (LPS) in mammary tissues caused by oxidative damage and inflammation has not been fully studied. In our experiments, we found that LPS significantly enhances oxidative stress by repressing the expression of oxidative-related factors and stimulating the secretion of inflammatory cytokines. Moreover, LPS decreases the body weight, water and feed intake and activates NF-κB, Jnk, Erk, and Nrf2. The relative protein intensity of P65 and pP65 were increased by LPS. However, resveratrol administration remarkably decreases the oxidative response as well as inflammatory genes and plays a vital role in protection of the mammary glands from negative alterations in female mice.

**Abstract:**

The aim of this study is to evaluate the defensive role of resveratrol, which is antagonistic to the oxidative stress and inflammation that is prompted by LPS in mammary tissue of female mice. Thirty adult mice were distributed into three groups (*n* = 10) control (CON), lipopolysaccharides at 2.5 mg/kg (LPS), and lipopolysaccharides at 2.5 mg/kg with 2 mg/kg of resveratrol (RES + LPS). The treatments were applied for 15 consecutive days. Spectrophotometry was used to quantify ROS in the blood, and proinflammatory cytokines concentrations were determined through radioimmunoassay. NF-κB, Jnk, IL-1β, Erk, IL-6, Nrf2 and TNF-α were quantified by RT-qPCR, and Western blots were used to quantifyP65 and pP65 protein intensities. MDA production was considerably increased, and the activity of T-AOC declined in the LPS treatment in comparison with the CON group but was significantly reversed in the RES + LPS group. Proinflammatory cytokines production and the genes responsible for inflammation and oxidative stress also showed higher mRNA and pP65 protein intensity in the LPS group, while Nrf2 showed a remarkable decline in mRNA expression in the LPS versus the CON group. All these mRNA intensities were reversed in the RES + LPS group. There were no remarkable changes in P65 protein intensity observed between the CON, LPS, and RES + LPS groups. In conclusion, resveratrol acts as a protective agent to modulate cellular inflammation and oxidative stress caused by LPS in mammary tissue of female mice.

## 1. Introduction

Lipopolysaccharides (LPS) are important molecules in a large, unique class of macromolecules. LPS are involved in the interaction of the cell with the environment. As a result, bacteria coming into contact with the immune system stimulate the production of certain antibodies that are primarily directed towards certain lipopolysaccharide structural components. Consequently, the primary surface antigens of Gram-negative bacteria are lipopolysaccharides [1]. A pathogen-associated molecular pattern (PAMP) based on circulating LPS can activate subsequently trigger a local or systemic inflammatory responses [2]. LPS is capable of triggering inflammation by activating cells other than immune cells. Such inflammatory cytokines can be closely monitored in an immune system response. The inflammatory pathways are the most essential part of innate immunity and possess the capability to trigger the complement system by the way of ROS pathways through the induction of endotoxins such as LPS [2]. The absence of LPS production by any given organism has long-term effects on the way other parts of the cell membrane are assembled [3]. Preeminent intensities of ROS activate inflammatory responses, trigger the NF-κB pathway, pro-inflammatory cytokines up-regulation and increase the production of ROS [4]. Gram-negative bacteria are an effective stimulator of inflammation at the outer membrane and have been frequently used as a hygienic sepsis model for lipopolysaccharide [5,6,7].

To inhibit ROS-induced injury, there is a large involvement of mammalian cells in the defense system of antioxidants. An enzyme called glutathione peroxidase (GPX) uses glutathione to neutralize dangerous lipid peroxides such as hydrogen peroxide [8]. Due to over production of antioxidant enzymes, oxidative stress occurs. Oxidative stress is the primary cause of several infectious diseases such as mastitis, pneumonia and enteritis in domestic animals, and earlier reports revealed that besides oxidative stress, inflammatory disease also occurs due to increased levels of nitric oxide (NO), ROS and Malon-dialdehyde (MDA) in serum [9,10]. In addition, oxidative stress is crucial for controlling metabolic processes in some organs and aids in maintaining or increasing domestic animal production [11]. In addition, dairy cows are under metabolic stress during lactation and pregnancy [12,13,14]. It is also thought that oxidative stress has a large impact on how productive farm animals are and how particular organs regulate their metabolism [15,16,17,18].

Most of the therapeutic compounds such as statins are well recognized for their roles in comprising immuno-modulatory and anti-inflammatory effects in addition to their innovative roles in disease treatment [19]. During stress conditions, herbal derivative compounds such as resveratrol (3, 5, 40-trihydroxy-trans-stilbene) have been shown to possess remedial effects [20]. Resveratrol has received attention because it possesses anti-inflammatory, anti-platelet aggregation, antioxidant, anti-atherogenic, anti-tumor and anti-aging activities [21]. Previously, resveratrol supplementation was found to be an effective for the treatment and prophylaxis of intestinal inflammation [22]. Previously, different compounds, such as sodium butyrate, have been shown to inhibit NF-κB signaling, which leads to an overall decrease in inflammation [23]. Resveratrol has been used to treat various diseases such as experimental colitis [24], pancreatitis [25] and arthritis [26]. The nutraceutical role of resveratrol, a phenolic compound, in combating the negative effects of LPS in mammary tissues caused by oxidative damage and inflammation have not been fully studied. Therefore, we propose to explore the key role of resveratrol in mitigating the harmful effects of lipopolysaccharides, such as oxidative damage, inflammation, and prompting the NF-κB/Nrf2 pathways, in the mammary tissue of a female mice model.

## 2. Materials and Methods

### 2.1. Ethical Approval

All experiments were conducted with the approval of The Institutional Animal Care and Use Committee (IACUC) which reviewed the experimental methods used in this research according to Department of Preventive Veterinary Medicine, Faculty of Veterinary and Animal Sciences, Ziauddin University Karachi.

### 2.2. Experimental Plan

In total, 30 adult (female) mice were purchased from the local market in Karachi, Pakistan. The mice were initially weighed (20–25 g) and equally distributed into three groups (*n* = 10). The first group was kept as a Control (CON) with no treatment. The second group was given lipopolysaccharides (2.5 mg/kg) LPS, and the third group was supplemented (2.5 mg/kg) lipopolysaccharides with resveratrol (2 mg/kg) RES+LPS. Standard balanced rodent feed in pellet form was provided to all mice (Jiangsu Synergistic Pharmaceutical Bioengineering Co., Limited, Nanjing, China). Bottles of water were replenished to maintain appropriate cleanliness and procedures. All the mice were allowed to adjust to the normal environmental conditions up to one week before the experiment was conducted. The feed intake, respiratory rate, and rectal temperature were observed daily throughout the trial. The mice were sacrificed for sampling on day 15.

#### Chemicals

Lipopolysaccharide (LPS, 0111: B4) and resveratrol were purchased from (Sigma Chemical Co., St. Louis, MO, USA). To investigate LPS-induced inflammation in mice, 2.5 mg/kg LPS was injected (intraperitoneal route) [27], and 2.0 mg/kg resveratrol was sequentially directed through oral gavage up to 15 days [28].

### 2.3. Determination of Body Weight, Feed and Water Intake in Each Group of Mice

Body weight (g), feed consumption (g) and the drinking of water (mL) were monitored continuously during the whole experimental period [29].

### 2.4. Biochemical Analysis of Oxidative and Antioxidative Biomarkersin Mammary Tissue

The antioxidant assays from mammary tissue were determined using commercially available assay kits for MDA, T-AOC, CAT, GPX and SOD, which were purchased from the Nanjing Jian Cheng Institute of Bioengineering. The analysis was also carried out in accordance with a previously published publication [18]. Briefly, 50 mg of frozen mammary tissue was homogenized for 30 **s** on ice at rpm in 10 mL of homogenization buffer consisting of 0.9% cool physiological saline. After subsequent centrifugation the supernatant was further diluted to different concentrations using physiological saline (0.9%), and the samples were stored at −20 °C for the analysis of various antioxidants as outlined below.

#### 2.4.1. Glutathione Peroxidase (GPX)

Glutathione peroxidase was analyzed spectrophotometrically (UV3600, Daojin Corp., Kyoto, Japan) at 412 nm using a commercially available kit (Nanjing Jiancheng Bioengineering Institute commercial kit, Nanjing, Jiangsu, China). To determine the decrease in GSH, the GSH’s rate of oxidation was measured within a set amount of time. 

#### 2.4.2. Superoxide Dismutase (SOD)

Superoxide dismutase was measured in the mammary tissue homogenate. A reactive system containing xanthine and xanthine oxidase makes up the SOD activity. Superoxide an ions oxidise hydroxylamine to produce nitrite at 550 nm, which was determined spectrophotometrically (UV3600, Daojin Corp., Kyoto, Japan). A sample’s ability to block the nitro blue tetrazolium reduction by 50% is considered to be one unit of SOD.

#### 2.4.3. Catalase (CAT)

Using a spectrophotometer (UV3600, Daojin Corp., Kyoto, Japan), catalase was detected in the breast tissue at 405 nm. According to a previous study by Wang et al. [30], the catalase activity often depends on the drop in H2O2 concentration in 15s. The breakdown of one MOL of H2O2 in one second constitutes one unit of catalase activity.

#### 2.4.4. Malondialdehyde (MDA)

Using commercially available kits, malondialdehyde concentrations were measured spectrophotometrically at 532 nm (Nanjing Jiancheng Bioengineering Institute, Nanjing, China). MDA activity was measured by a previously described technique, the thiobarbituric acid reaction method described by Chen et al. [31].

#### 2.4.5. Determination of Total Antioxidants (T-AOC)

Total antioxidant concentration (T-AOC) was measured using spectrophotometry. The reduction acts together with phenanthrene to produce a colored molecule that can be seen at 520 nm. The optical density is raised by 0.01 units of T-AOC per milligram of protein.

### 2.5. Radioimmunoassay

Plasma samples of mice were separated from blood, frozen and stored at −20 °C. IL-1, IL-6, and TNF-a concentrations were evaluated using radioimmunoassay kits according to manufacturer’s instructions (Beijing North Institute of Biological Sciences, Beijing, China).

### 2.6. Real-Time (qRT-PCR)

#### Total RNA Isolation and Quantitative Real-Time PCR

Each sample was homogenized with Tryzol for 15s and analyzed by absorption at 260 nm. The RNA was quantified by agarose gel electrophoresis and by nano-drop with spectrophotometer (Eppendorf Biotechnology, Hamburg, Germany). Samples with a ratio of A260-A280 and in a range of 1.8 to 2.1 were used in these experiments. First-strand cDNA was primed with a commercial kit, the Prime Script RT Master Mix Perfect Real Time Kit (Takara Co., Otsu, Japan) according to the manufacturer guidelines.

To quantify NF-κB, IL-1β, Jnk, IL-6, Erk, TNF-α and Nrf2 transcripts, the primer sequence for the specified genes and β-actin as a reference gene were derived from a previous study [31,32,33,34]. All the analyses were conducted in triplicate, and a gel electrophoresis was performed to confirm the particular PCR products on a 3.0% agarose gel. A detailed procedure for qRT-PCR was followed as described in previous research [2].

### 2.7. Western Blot Analysis

Total protein was isolated from 100 mg of glandular tissue sample and quantified by the Bradford protein assay (Bio-Rad, Hercules, CA, USA). Centrifugation at 14,000 rpm for 15 min was used to create the supernatant. A bicinchoninic acid (BCA) protein assay kit was used to measure the amount of protein in the sample (Beyotime, Shanghai, China). A 30 g sample of protein was electrophoretically separated on a 10% Bis-Tris Nu-PAGE gel before being transferred to PVDF membranes. The gel was transferred into the nitrocellulose membranes by means of a 100 V wet transfer device for 60 min after the electrophoresis process. The membrane blocking was performed for 2h in 10% bovine serum albumin (BSA) or 5–10% skimmed milk diluted in 1*TBST, followed by primary antibodies incubation (1:1000 dilution) at 4 °C for 12 to 14 h. The membranes were then washed thrice with TBST and incubated with a secondary antibody (1: dilution anti-rabbit or anti-goat IgG) conjugate-(HRP) at room temperature for 2 h. The washing step was repeated thrice, and the proteins were detected by chemiluminescence [35].

### 2.8. Statistical Analysis

One-way ANOVA was utilized for grouped analysis. Deviations were considered significant when *p <* 0.05. The visualization was created using Graph Pad 8.0 software (CA, USA). * *p <* 0.05, ** *p <* 0.005 LPS vs. CON.

## 3. Results

### 3.1. Comparison of Anthropometric Parameters

**Figure 1:**
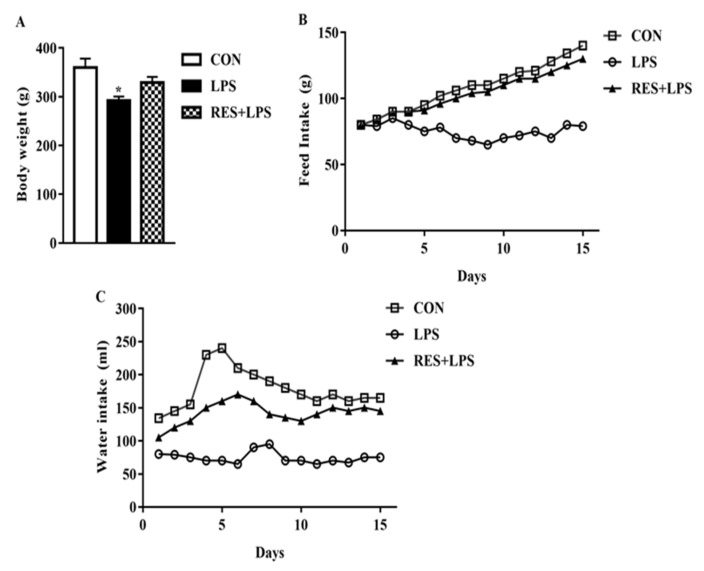
(**A**) Body weight, (**B**) feed intake, and (**C**) water intake of Control (CON), Lipopolysaccharide (LPS) and Resveratrol (RES) groups. Levels were significantly decreased in the LPS-fed group compared with the CON and RES+LPS groups. No significant difference was observed for the CON vs. LPS+RES group comparison. * *p <* 0.05, LPS vs. CON.

The mice body weight in LPS was markedly different from CON and RES groups, as described in Figure 1A. Variation in the food and water intake was observed in the LPS and RES groups in contrast to the CON group throughout the experimental period of 15 days (Figure 1B,C).

### 3.2. MDA Production and T-AOC Capacity in the Plasma Samples of Female Mice

**Figure 2:**
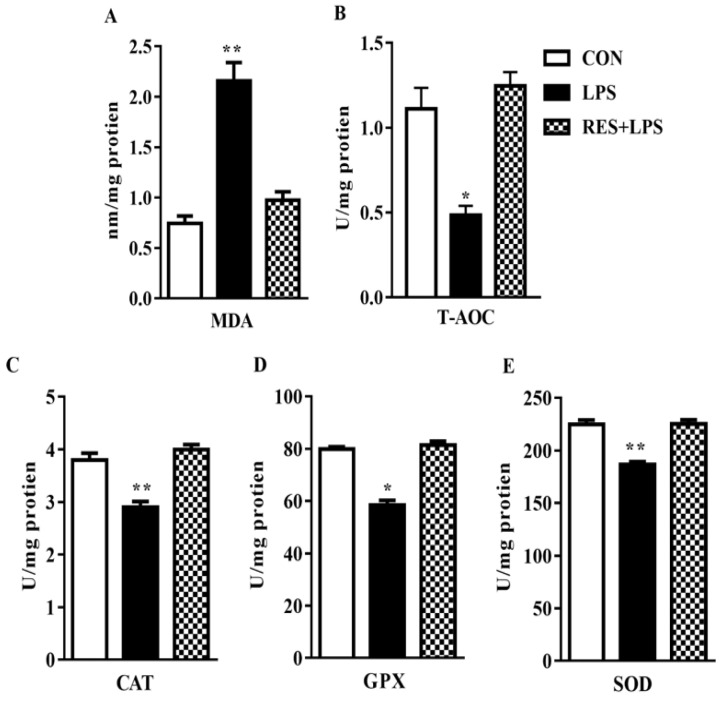
The effect of resveratrol on antioxidant enzyme activity and total antioxidant capacity (**A**) Malondialdehyde (MDA) (**B**), Total Antioxidants (T-AOC) (**C**), Catalase (CAT) (**D**), Glutathione Peroxidase (GPX) and (**E**) Superoxide Dismutase (SOD) enzyme activities in the mammary tissue of female mice. * *p <* 0.05 significant and ** *p <* 0.005 highly significant.

Malondialdehyde (MDA) level increased dramatically (*p* < 0.002; Figure 1A), whereas total antioxidant capacity (T-AOC) substantially declined (*p* < 0.04; Figure 1B) in the mammary tissue samples of mice in comparison with the CON group. Ultimately, these changes were totally reversed by resveratrol, as observed in the RES group in contrast to LPS. No significant changes were observed between the CON or RES groups (Figure 2).

### 3.3. Proinflammatory Cytokines Concentration in the Plasma Samples

**Figure 3:**
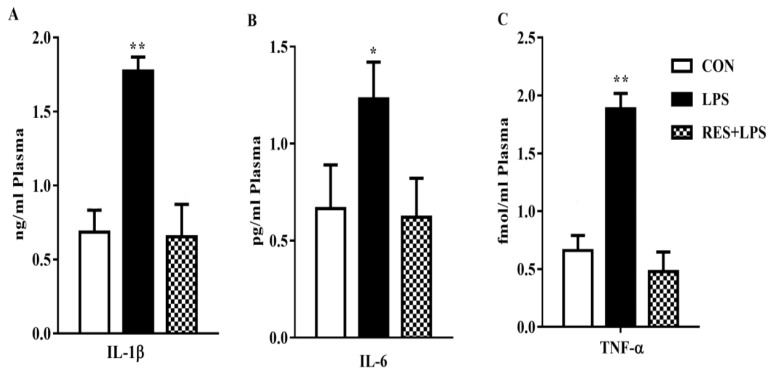
Concentration of proinflammatory cytokines in mammary tissue of female mice: (**A**) IL-1β, (**B**) IL-6, and (**C**) TNF-α. * *p <* 0.05 significant and ** *p <* 0.005 highly significant.

The concentrations of proinflammatory cytokines in plasma samples were significantly increased in the LPS group in comparison with the CON group. However, these increased levels were reversed by resveratrol, as can be observed in the RES group vs LPS comparison (Figure 3).

### 3.4. The mRNA Levels of Proinflammatory Cytokines in Mammary Tissue of Female Mice

**Figure 4:**
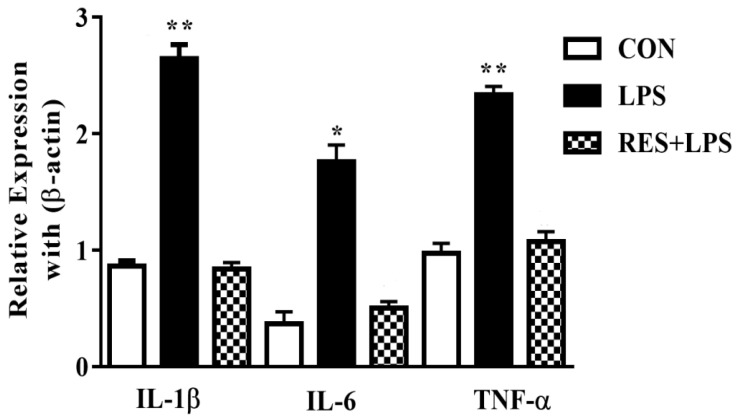
The mRNA expressions of proinflammatory cytokines (IL-1β, IL-6 and TNF-α) in mammary tissue of female mice. * *p <* 0.05 significant and ** *p <* 0.005 highly significant.

The mRNA expressions in mammary tissues were substantially upregulated by LPS in comparison with the CON group. Meanwhile, these mRNA levels were reversed by resveratrol, as observed in the comparison between the RES and LPS groups (Figure 4). 

### 3.5. The mRNAs Expression of NF-κB, JNK, ERK and Nrf2 in Mammary Tissue of Female Mice

**Figure 5:**
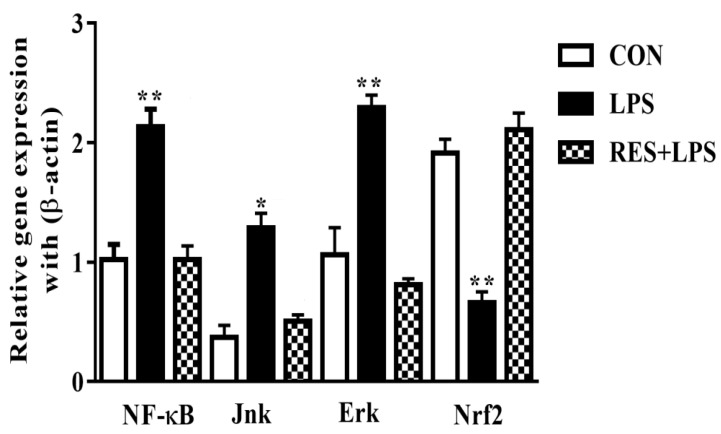
The mRNA expressions of NF-κB, Jnk, Erk, and Nrf2 in mammary tissue of female mice. NF-κB, JNK, and ERK levels were significantly different in the LPS group, while the Nrf2 mRNA expressions were significantly different in the LPS group in contrast to the CON and LPS+RES groups. * *p <* 0.05 significant and ** *p <* 0.005 highly significant.

The mRNA levels of adaptor/inflammatory molecules were remarkably increased by LPS in comparison with the CON and RES groups. However, the Nrf2 mRNA expressions (*p <* 0.002) were completely opposite in the LPS group compared with the CON and RES+LPS groups. The expressions of NF-κB and Nrf2 were dramatically reversed by resveratrol, as observed in RES in comparison with the LPS group. No significant differences were observed between the RES and CON groups (Figure 5).

### 3.6. The Protein Expression of NF-κB (P65 and PP65) in Mammary Tissue

**Figure 6:**
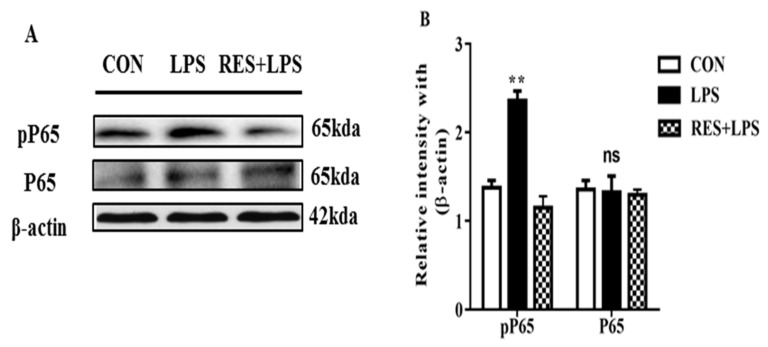
(**A**) Immunoblots and (**B**) quantification of pP65 and P65 in LPS in comparison with CON and RES+LPS groups. ns: not significant and ** *p <* 0.005 highly significant.

The pP65 (*p* < 0.003) protein intensity was remarkably increased in the LPS in comparison with the CON groups. Additionally, this intensity of P65 (*p <* 0.6) was significantly reduced in the RES group. However, the P65 intensity remained similar, and no remarkable changes were found in any of the groups (Figure 6A,B).

## 4. Discussion

Lipopolysaccharide (LPS), a substance found in the outer membranes of Gram-negative bacteria, triggers several significant cellular reactions that are vital to the pathophysiology of inflammatory responses. An immediate inflammatory response to pathogens may result from LPS. As bacterial LPS causes a variety of cell types to generate inflammatory cytokines, including IL 6 and IL 1, it has been widely employed to create an inflammatory model. [31]. It has been observed that ROS plays a dynamic role in mastitis and its pathophysiology [36], in lung injury, and in cancers [37]. Pathogenic bacterial infections activate immune responses in host cells during mastitis, which leads to ROS production [38]. As reported previously, disproportionate amounts of ROS may cause cellular damage because of lipid oxidation [39]. Detection of MDA in plasma as an effect of oxidative lipid damage can provide comprehensive evidence about lipid oxidation, and higher levels of MDA production parallel to an imbalance of oxidants and antioxidants [40]. Consistent with a previous study, we also observed higher MDA production and T-AOC subordination in the LPS group [41]. It is clear that the augmented Nrf2 and its interrelated antioxidant levels could be linked to an adaptive response in the body prompted by LPS. Nrf2 is an important transcription factor as it sets the expression of several genes such as detoxification and antioxidant genes. When activated, it can increase the production of a variety of antioxidant enzymes and proteins, including glutathione, catalase, and superoxide dismutase, which can help protect cells from oxidative stress and inflammation. The innate immune system is highly boosted by LPS, which is detected by TLR4 and causes the immune system to respond. Auxiliary molecules such as LPS binding protein (LBP) and differentiation group 14 contribute to the recognition of LPS TLR4 (CD14) [42].The primary transcription factor which controls the production of cytokine genes is NF-κB.NF-κB is frequently present in cytoplasm with the inhibitor κB in an inactive form. It is released from NF-B-IκB after it has been phosphorylated [43].

In response to LPS being present in the blood, the immune system can trigger an immune response. This response may involve activation of immune cells which recognize and respond to LPS via specific receptors called toll receptors (TLRs) [44].A higher level of ROS causes release of NF-κB and increases cytokine concentration to initiate inflammatory responses [4,45]. Here, we found that NF-κB, JNK, ERK and pro-inflammatory cytokine mRNA levels were remarkably increased in the LPS group, an effect which was predominantly reversed by resveratrol, as indicated by the RES group data. As shown previously, during the inflammatory process, various transcriptional molecules, e.g., NF-κB, AP-1 and MAPK, are strongly regulated in a redox-dependent manner [46,47]. TNF is the first endogenous mediator of an inflammatory reaction and is essential for the acute phase response of inflammation as successive inflammatory reactions drive mammary epithelial cells to produce more TNF- and IL-1 [45]. These inflammatory factors can serve as indicators of the degree of inflammation. The amount of proinflammatory cytokines was remarkably higher in the LPS group, and these changes were reversed by resveratrol in the RES group. This contrasts with the view that resveratrol supplementation is a prospective approach against intestinal inflammation therapy because it acts as an anti-allergic, antioxidant and anti-inflammatory agent [21,22,28]. The increased number of cells such neutrophils, macrophages, and differentiated cells are some of the significant factors that contribute to the copious production of free radicals in mastitis milk [48]. Increased levels of TNF-, IL-1b, IL-6, and (NO) nitric oxide are another contributing cause [49]. Reactive nitrogen intermediates (RNI), which are involved in the complex process of inflammation, are activated by cytokines [50].

To maintain tissue integrity, Nrf2 dynamically defends cellular antioxidants against ROS [51]. In the regulation of target regions, Nrf2 binds with antioxidant response element (ARE) due to oxidative damage and activates several cytoprotective genes and antioxidants [52]. Decreases in Nrf2 and the mRNA concentrations of other antioxidants in the LPS group indicate suppression of the antioxidant system. Our results revealed that the downregulation of Nrf2 is because of higher amounts of oxidative stress, as Nrf2 protects cell and tissue damage, whereas a previous report found that P38 and JNK yield stress cause inflammation [43]. Moreover, Zhang et al. also revealed that stimulation of signaling pathways such as MAPK is associated with inflammatory effects and stress on the mammary glands of cows. When activated, MAPK can lead to increased cell proliferation and differentiation, which can be beneficial for mammary gland development and function [53]. It has been also reported that NF-κB is an LPS-mediated pro-inflammatory cytokines activator [54,55]. We also investigated the protein intensity of NF-κB. The protein density of pP65 was remarkably higher in LPS compared with the CON group. Additionally, the density of PP65 was significantly decreased in the RES group. All the groups showed no remarkable changes in P65 density. In this research, we have uncovered the defensive role of resveratrol in combating the adverse influences of LPS, such as the induction of oxidative damage and inflammation in the mammary tissue of female mice.

## 5. Conclusions

Taken together, our results reveal that resveratrol acts as a protective agent to modulate LPS-induced cellular injury and oxidative stress responses in the mammary tissue of female mice. This protection mainly occurs by combating the NF-κB and MAPK mechanisms, by Nrf2 signaling activation, thereby decreasing the proinflammatory cytokine production and improving the antioxidant levels. Importantly, uncovering the detailed mechanisms of action of resveratrol in the LPS-induced inflammatory animal model is essential in the future.

## Data Availability

Not applicable.
